# Effects of Ayahuasca on Personality: Results of Two Randomized, Placebo-Controlled Trials in Healthy Volunteers

**DOI:** 10.3389/fpsyt.2021.688439

**Published:** 2021-08-06

**Authors:** Juliana Mendes Rocha, Giordano Novak Rossi, Flávia L. Osório, José Carlos Bouso Saiz, Gabriela De Oliveira Silveira, Mauricio Yonamine, Eduardo José Crevelin, Maria Eugênia Queiroz, Jaime E. Cecílio Hallak, Rafael Guimarães Dos Santos

**Affiliations:** ^1^Department of Neurosciences and Behavior, Ribeirão Preto Medical School, University of São Paulo, Ribeirão Preto, Brazil; ^2^National Institute of Science and Technology—Translational Medicine, Ribeirão Preto, Brazil; ^3^International Center for Ethnobotanical Education, Research and Services, ICEERS Foundation, Barcelona, Spain; ^4^Medical Anthopology Research Center, Universitat Rovira i Virgili, Tarragona, Spain; ^5^School of Pharmaceutical Sciences, University of São Paulo, São Paulo, Brazil; ^6^Departamento of Chemistry, University of São Paulo, Ribeirão Preto, Brazil; ^7^School of Pharmaceutical Sciences, Ribeirão Preto Medical School, University of São Paulo, Ribeirão Preto, Brazil

**Keywords:** ayahuasca, dimethyltryptamine, hallucinogens, psychedelics, personality

## Abstract

**Rationale:** Previous studies with the serotonergic hallucinogens LSD and psilocybin showed that these drugs induced changes in personality traits, such as increases in Openness. However, results are inconsistent, and the effects of ayahuasca on personality were never investigated in a controlled trial.

**Objectives:** To assess the effects of ayahuasca on personality in two randomized, placebo-controlled trials in healthy volunteers.

**Methods:** Data from two parallel-group, randomized, placebo-controlled trials in healthy volunteers were included. In the first trial, 15 volunteers ingested ayahuasca or placebo, while in the second trial 15 volunteers received placebo+ayahuasca or cannabidiol (CBD)+ayahuasca. Personality was assessed with the NEO-Five Factor Inventory (NEO-FFI) at baseline and 21 days post-treatment.

**Results:** There were significant differences between groups in baseline Openness scores, but not on day 21. A significant increase in Openness scores was observed in the placebo + ayahuasca group in study 2. No other within-group differences were observed for any other domain.

**Conclusions:** Ayahuasca produced inconsistent effects on personality since it induced significant increase in Openness 21 days post-drug intake only in one of the trials. The absence of significant differences in the other ayahuasca groups could be due to small sample sizes and baseline differences among groups. The effects of ayahuasca and other serotonergic hallucinogens on personality should be further investigated in clinical samples.

## Introduction

The relationship between personality and serotonergic hallucinogens such as lysergic acid diethylamide (LSD), psilocybin, and ayahuasca/dimethyltryptamine (DMT) is poorly understood (1). A recent systematic review suggested that these drugs can induce changes in personality traits, such as Openness to experience and Self-transcendence (1, 2). Specifically, in a pooled analysis of two placebo-controlled trials involving administration of single and multiple doses of psilocybin to healthy volunteers a significant increase in the Openness to experience factor of the NEO-Five Factor Inventory (NEO-FFI) was observed 14 months following a high-dose psilocybin session (3). However, results from the original trial (4) were non-significant, and the doses used in the trials (single vs. multiple) were different. A non-placebo-controlled trial with healthy volunteers corroborated the increase in Openness 6 months after psilocybin (5), but a recent placebo-controlled trial from the same group failed to find significant effects in Openness 1-month post-psilocybin, finding only a significant increase in Conscientiousness (6). In a recent open-label trial involving two doses of psilocybin in patients with treatment-resistant depression, significant increases in Openness and Extraversion and significant decreases in Neuroticism were observed at the 3-month follow-up (7). Regarding LSD, one placebo-controlled study in healthy volunteers found significant increases in Openness 2 weeks post-LSD (8). Another placebo-controlled trial did not find significant effects on Openness one and 12 months after LSD administration, finding only a significant increase in Conscientiousness (9).

Regarding ayahuasca (a hallucinogenic botanical brew rich in β-carboline alkaloids and DMT), observational studies of ritual ayahuasca users reported increases in Openness (10) and Self-transcendence (a personality trait related to Openness) after continuous use (11). However, to the best of our knowledge, the effects of ayahuasca on personality were never assessed in a controlled trial. Additionally, parallel-designs including a control group in addition to the psychedelic are mostly lacking for all classic hallucinogens. Thus, the current article presents data from two randomized, double-blind, placebo-controlled clinical trials that evaluated the effects of ayahuasca on personality traits in healthy volunteers. Considering the above-mentioned evidence of associated changes in personality in other classic hallucinogens, and the antidepressive and anxiolytic effects of ayahuasca, our hypothesis was that ayahuasca would produce increases in Openness and Conscientiousness scores and decreases in Neuroticism scores.

## Materials and Methods

### Volunteers

Study 1 was conducted from November 2017 to May 2019 and had as the primary outcome the effects of ayahuasca on emotion face recognition, and as secondary outcomes subjective effects, safety, and personality measures [the results were recently published (12). Study 2 was conducted from September 2018 to March 2020 and had as the primary outcomes the effects of ayahuasca on emotion face recognition and empathy, and as secondary outcomes subjective effects, safety, and personality measures (manuscript in preparation).

In study 1, 49 participants were screened through the Structured Clinical Interview for Mental Disorders (SCID-5-CV, DSM-5) via telephone (13) with an experienced psychologist to verify eligibility for participation in the study. After the interview, 22 participants were included in the study, with 11 randomized to the placebo group and 11 in the ayahuasca group. One volunteer completed the experimental session but was excluded from further analysis in the ayahuasca group due to loss of baseline data for an emotion face recognition task (primary outcome in the original trial). Another volunteer was excluded in the placebo group due to hypoglycemia at the last blood collection point in the experimental session. Therefore, 20 volunteers were included in the final sample (10 per group). The mean age was 31.8 years (range 21–55), and the mean weight was 72.8 kg (range 53–101) [see details in (12)]. Of these 20 volunteers, personality data for day 21 was lost for five volunteers (errors and/or unanswered items). Therefore, 15 volunteers were included in the final personality analysis of study 1 (eight in the ayahuasca group and seven in the placebo group). In study 2, 35 participants were screened using the same procedures of study 1 (SCID-5-CV), and 17 were included in the trial. Nine volunteers were randomly included in the cannabidiol (CBD) plus ayahuasca group and eight in the placebo plus ayahuasca group. The mean age was 25.3 years (range 20–36), and the mean weight was 68.1 kg (range 49–110). All volunteers completed the experimental sessions. However, two volunteers were excluded in the placebo plus ayahuasca group due to home use of ayahuasca bought on the Internet 2 weeks after their experimental session. Thus, 15 volunteers were included in the final personality analysis (nine in the CBD plus ayahuasca group and six in the placebo plus ayahuasca group). The flowchart for study participants in both trials is shown in the Online Resource 1.

In both trials, volunteers were invited through contact with researchers or other study participants. Eligibility criteria in both trials included age between 18 and 65 years. However, studies differed regarding drug use. In study 1, eligibility criteria included the absence of prior use of ayahuasca and ≤ 2 uses in life of other hallucinogens (LSD, psilocybin, DMT, mescaline, other tryptamines and phenethylamines). In study 2, eligibility criteria included ≤ 2 lifetime uses of hallucinogens (including ayahuasca) and ≤ 20 lifetime uses of other recreational drugs. In both studies, exclusion criteria included a current or past history of cardiovascular, liver and/or neurological diseases, any psychiatric diagnosis (SCID-5), use of psychoactive medications (antidepressants, mood stabilizers, anxiolytics and antipsychotics), recurrent use (> twice a month) of drugs of abuse (cannabis and cocaine confirmed by urinalysis and other drugs as reported by volunteers), and pregnant or lactating women.

Groups were homogeneous in terms of sociodemographic characteristics (the sociodemographic characteristics of the volunteers are shown in the Online Resource 2). Regarding clinical characteristics, all volunteers included in both trials were considered healthy, that is, none had a history of mental disorders, chronic diseases, or other clinical conditions. In addition, none of the participants were using psychotropic medications. In study 1, none of the volunteers had a history of drug use (including ayahuasca and other hallucinogens). In study 2, volunteers had a mean lifetime use of cannabis of 7.3 uses (range 2–20), with the last use at least 1.5 months (mean 15.3 months) before the trial. Cocaine was used by only one volunteer, 4 years before the trial. Methylenedioxy methamphetamine (MDMA) was used by four volunteers a mean of 3.75 lifetime uses (range 1–6), with the last use at least 3 months (mean 7.25 months) before the trial. LSD was used once by one volunteer, 6 months before the trial. Ayahuasca was used by three volunteers, with a mean of 1.3 lifetime uses (range 1–2), with the last use at least 6 months before the trial (mean 20 months). Solvents were used by two volunteers with a mean of 7.5 lifetime uses (range 5–10), with the last use at least 5 months before the trial (range 5–48 months).

The trials were approved by the Research Ethics Committee of the Hospital das Clínicas, Faculty of Medicine of Ribeirão Preto, University of São Paulo (HC-FMRP-USP). All procedures were conducted in accordance with the Declaration of Helsinki and the ethical standards of the Ministry of Health (Resolution No. 466/12 of the National Health Council). Volunteers received detailed information on the nature of ayahuasca, the general psychological effects of hallucinogens/psychedelics and their possible adverse effects, as reported in the psychiatric literature. All volunteers gave their written informed consent to participate.

### Drugs

In study 1, the administered drugs were placebo and ayahuasca (both 1 mL/kg). In study 2, the treatments were 600 mg CBD (99% purity diluted in sunflower oil, administered in two 00 capsules containing 300 mg of CBD each; BioSynthesis Pharma Group Ltd, United Kingdom) plus ayahuasca, and placebo plus ayahuasca (ayahuasca dose of 1 ml/kg). The ayahuasca dose administered was chosen based on previous work by our group in which it caused psychotropic effects (14–16). The ayahuasca batches used during the study were donated by the *Santo Daime* church *Rainha do Céu* (“Queen of heaven”) (Ribeirão Preto, Brazil), which uses ayahuasca in their religious rituals. The religious use of ayahuasca is permitted in Brazil, as well as for scientific research. Ayahuasca was prepared via the prolonged decoction of the bark of the *Banisteriopsis caapi* vine (rich in the β-carbolines harmine, tetrahydroharmine/THH, and harmaline) together with the leaves of the *Psychotria viridis* bush (rich in DMT). The Brazilian legislation demands that ayahuasca must be made using only these two species, but the quantity of each plant used, and the time of decoction vary substantially from group to group. The ayahuasca batch in study 1 was prepared in October 2017, and the batch used in study 2 was prepared in July 2018. After preparation, ayahuasca was cooled and stored according to the religious institution guidelines (at room temperature protected from light) until the first experimental session (November 2017 in study 1 and September 2018 in study 2). Alkaloid stability was assessed in study 1 from November 2017 until the end of the trial 18 months later (May 2019), and from September 2018 until the end of the trial 16 months later (March 2020) in study 2, using ultra-performance liquid chromatography-electrospray tandem mass spectrometry (UPLC-ESI-MS/MS) [see [12] for detailed method description]. In study 1, the average concentration and variation of alkaloids was 1.58 (range: 0.45–3.04) mg/mL for DMT, 1.15 (range: 0.30–1.57) mg/ml for harmine, 0.73 (range: 0.59–0.81) mg/ml for THH, and 7.38 (range: 2.07–11.80) mg/ml for harmaline. In study 2, the average concentration and variation of alkaloids was 0.67 (range 0.45–1.00) mg/ml for DMT, 0.87 (range: 0.30–1.50) mg/ml for harmine, 0.52 (range: 0.35–0.80) mg/mL for THH, and 0.05 (range: 0.03–0.57) mg/ml for harmaline. Considering previous evidence of dose-dependent effects of ayahuasca [especially regarding DMT; (17)], these alkaloid differences seem to be relevant and, therefore, could influence the results.

In study 1, the placebo was a non-psychoactive substance intended to produce in the participant the sensation of drinking a nauseous and bitter medicine. Since all volunteers were naive to the use of ayahuasca in this study, they did not know the organoleptic properties of ayahuasca. Placebo was prepared with substances that are commonly used as food additives and in pharmaceutical formulations: mineral water (500 mL), glycerin 5% (E422), purchased from Galena (Campinas, SP, Brazil), propylene glycol 5% (E1520), purchased from Fagron (São Paulo, SP, Brazil), and methylparaben 0.1% (E218), purchased from Ely Martins (Cravinhos, SP, Brazil). For study 2, where the blinding procedure was done regarding CBD administration, the placebo was two 00 capsules identical to the ones that contained CBD plus sunflower oil, but with just the oil. Ayahuasca and placebo were administered in brownish/opaque glass bottles of 200 mL so that participants and researchers could not see the color of the liquid. Moreover, in the experimental session volunteers were instructed to ingest the full content of the bottle without smelling or visually examining its content. To avoid an accidental identification of the substance via smell by the authors in study 1, volunteers were instructed to open the bottle and drink the contents at least two meters away from researchers.

### Study Design

Both studies used a randomized, double-blind, placebo-controlled, parallel-group design. Simple randomization was performed in both trials by a researcher who did not participate directly in the experimental sessions and did not have access to the raw data of the study. We recommended abstention from alcohol, tobacco, and caffeinated drinks 24 h prior to the experimental sessions. Volunteers were instructed to fast before the session and to not ingest tyramine containing foods/drinks 24 h before and 12 h after the experimental session to avoid possible interactions with the β-carbolines in ayahuasca, which are reversible inhibitors of the monoamine oxidase enzyme type A (MAO-A). It was suggested for volunteers to wear comfortable clothes and to avoid using cell phones/social media during the experimental sessions. Reading was allowed (we offered some magazines with themes related to nature and science, but volunteers could bring their own reading). Music and movies were not allowed so that the environment during the effects of ayahuasca was kept as quiet and neutral as possible.

No specific psychotherapeutic intervention was used before, during, or after the experiments, as in our previous studies (14–16). Our protocol included general principles described in the psycholytic and psychedelic models of the 1960–1970s and in recent guidelines for safety in human hallucinogen research (18). These include (i) creation of trust with the volunteers before the experiment, (ii) use of a non-directive, supportive approach during drug sessions, and (iii) follow-up encounters from some days to several weeks after the session. There were no pre-dose visits, and the volunteers encountered the personnel directly involved in the trial (JMR, GNR, RGS) only in the day of the experimental session. Before the session, volunteers were informed about the general effects of ayahuasca and could express any doubts about the experimental session. During the sessions, simple instructions were given: “remain as quiet and introspective as possible, with your eyes opened or closed, while focusing on your body, thoughts, and emotions.” Volunteers remained seated in a comfortable reclining chair in a quiet dimly-lit room. Researchers stayed in a room next to the room where the volunteers were and assessed their well-being during the data collection points. Researchers only remained with volunteers in cases when it was requested by the volunteer or if researchers judged it necessary due to the intensity of psychoactive effects. At the end of the experimental session (4 h after ayahuasca intake), the general condition of the volunteers was evaluated, and if the volunteers were feeling well, they were discharged.

In the days to weeks after the experimental session, volunteers freely described their experiences, but no specific integration technique was used. In study 1, follow-ups occurred 1, 7, 14, and 21 days and 3 months after drug intake [see (12) for results not related to personality]. In study 2, follow-ups occurred 1, 7, and 21 days after drug intake (results not related to personality in preparation). In both trials, personality was assessed at day 21. Follow-up encounters were standardized for both studies.

Volunteers were offered R$ 20.00 (~$4.00) for going to the laboratory in the experimental and follow-up sessions.

### Study Methods

#### NEO-FFI-R

Personality was assessed and evaluated by a trained psychologist using the Brazilian version of the NEO-FFI-R instrument (19), which covers five domains: Neuroticism, Extraversion, Openness to Experience, Conscientiousness, and Agreeableness. This version has 60 items (12 per domain) that are rated by respondents using a five-point Likert-type scale ranging from “strongly disagree” to “strongly agree.”

#### Statistical Analyses

Since there is no previous study assessing the effects of ayahuasca on personality and both trials were pilot studies in which personality was not the primary outcome, no formal sample size calculation was performed. NEO-FFI-R subscales raw scores were transformed according to the technical instructions and were analyzed using a 2-way repeated-measures analysis of variance (ANOVA) with Time (number of assessments) as the within-subject factor and Group (ayahuasca, placebo, placebo + ayahuasca, CBD + ayahuasca) as the inter-subject factor. All the necessary tests to meet the statistic criteria (normality and homoscedasticity) were carried out. Statistical significance was used at *p* < 0.05 corrected for multiple comparisons using the Bonferroni test. The effect size was calculated using η^2^. The statistical program SPSS (version 21) was used.

## Results

**Figure 1:**
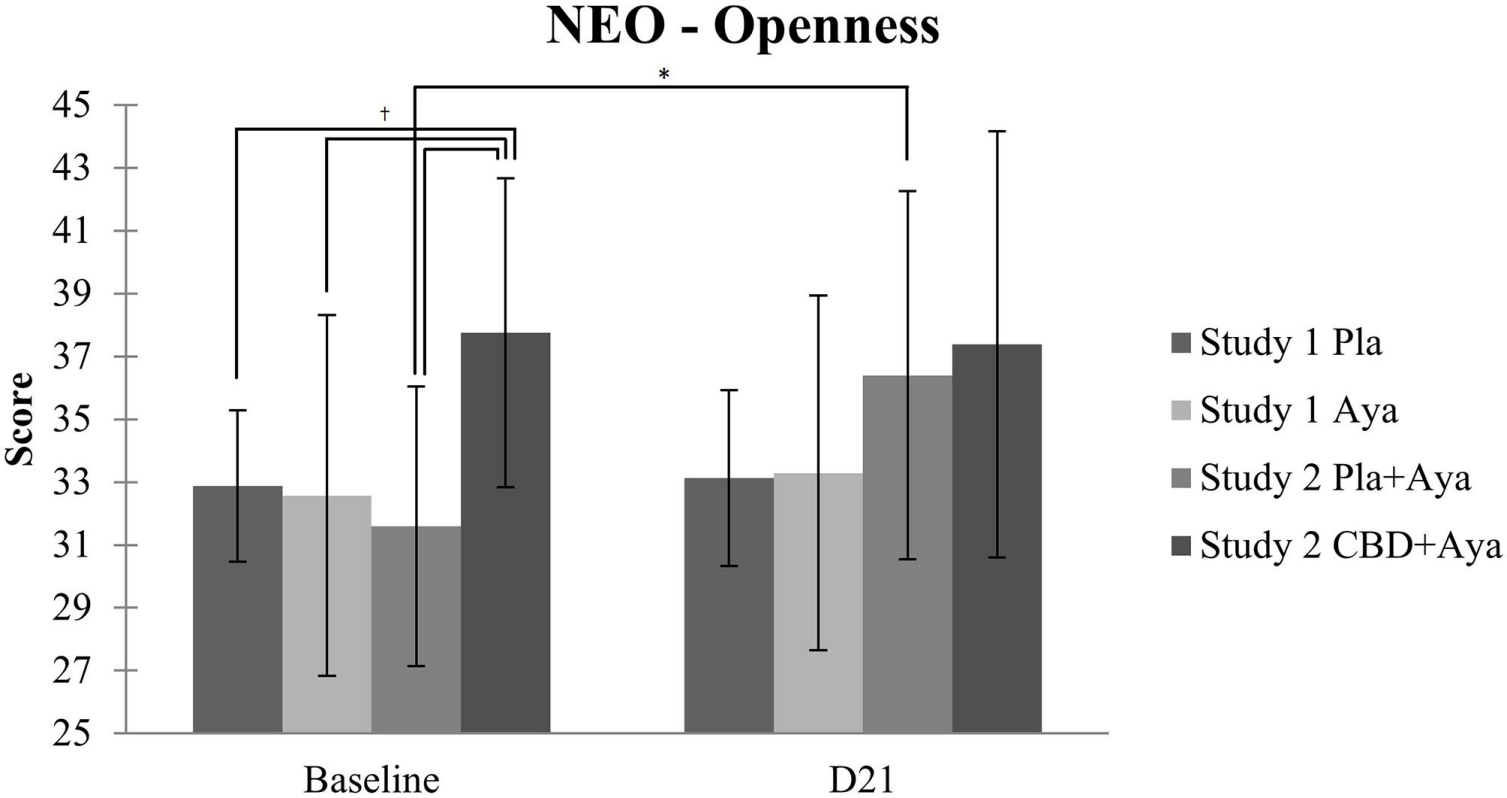
Openness to experience scores (mean; SD) on the four groups between baseline and day 21 (D21): placebo and ayahuasca (study 1, *n* = 15), and CBD plus ayahuasca and placebo plus ayahuasca (study 2, *n* = 15). Significant differences were observed between groups at baseline (*p* < 0.05, corrected) and in the placebo plus ayahuasca group in study 2 from baseline to D21 (*p < 0.05, corrected).

There were no significant effects of Time or in the Time x Group interaction in Neuroticism, Extraversion, Conscientiousness, and Agreeableness in both trials (*p* > 0.05) (mean scores for all domains in shown in the Online Resources 3, 4). Regarding Openness, there was a significant effect of Time [*F*_(1, 24)_ = 5.749, *p* = 0.025, η^2^ = 0.192] and in the Time x Group interaction [*F*_(3, 24)_ = 3.595, *p* = 0.028, η^2^ = 0.310]. In the comparison between groups, baseline differences were found between the CBD plus ayahuasca group (mean 37.75, SD = 4.92) and the ayahuasca group (mean 32.57, SD = 5.74) (*p* = 0.041), the placebo group (mean 32.88, SD = 2.41) (*p* = 0.036), and the placebo plus ayahuasca group (mean 31.60, SD = 4.45) (*p* = 0.025) (Figure 1). No significant differences were found between groups on day 21 (*p* > 0.05). In study 2, there was a significant increase in Openness scores when comparing baseline with day 21 (from 31.60 to 36.40, *p* = 0.01) in the placebo plus ayahuasca group.

## Discussion

In this analysis of changes in personality from two randomized controlled trials involving the administration of ayahuasca to healthy volunteers, no significant effects were observed in Neuroticism, Extraversion, Conscientiousness, and Agreeableness scores within and between groups. The only significant results were observed in Openness, where groups were significantly different on baseline scores, and a significant increase in Openness scores 21 days after drug intake compared to baseline was observed in the placebo plus ayahuasca group of study 2.

Previous studies with psilocybin and LSD have reported inconsistent results on personality measures. In healthy volunteers, a pooled analysis of two placebo-controlled trials showed a significant increase in Openness scores 14 months following a high-dose psilocybin session (3). However, results from the original trial (4) and from a more recent study (6) were nonsignificant for this personality domain. On the other hand, a non-placebo-controlled study by the same group reported increases in Openness 6 months after the last drug dose (5). In a recent open-label trial involving two doses of psilocybin to patients with treatment-resistant depression, significant increases in Openness were observed at the 3-month follow-up (7). Regarding LSD, one placebo-controlled trial with healthy volunteers found significant increases in Openness 2 weeks post-LSD (8), while another placebo-controlled trial did not find significant effects one and 12 months after LSD intake, reporting only a significant increase in Conscientiousness (9).

The inconsistencies found in other studies were also observed in the present investigation. Firstly, only in study 2 the administration of ayahuasca was associated with a significant increase in Openness. The same outcome was not observed in volunteers who took ayahuasca in study 1 (compared to placebo). Considering that both studies were carried out in the same set and setting and in healthy volunteers, these conflicting results could be accounted for by a lack of statistical power to demonstrate a statistically significant difference and perhaps by differences in study populations and/or in the alkaloid composition. For instance, participants in study 2 had a history of illicit drug use, perhaps reflecting “unconventional values” (a facet of openness) that was particularly susceptible to the ayahuasca psychedelic experience. Moreover, since the CBD plus ayahuasca group had the highest Openness scores at baseline, it is possible that further rises in this trait were not verified because these volunteers already had an above-average Openness trait. This could have inhibited ayahuasca's effect on this variable due to a ceiling effect. Regarding alkaloid composition, previous controlled trials showed evidence of dose-dependent effects of ayahuasca, especially regarding DMT (17). We observed a relevant variation in the average concentration of alkaloids both within and between studies, including for DMT [study 1: 1.58 (range: 0.45–3.04) mg/ml; study 2: 0.67 (range 0.45–1.00) mg/ml]. These alkaloid differences could have influenced the observed results. Additional reasons to explain differences between our trials and previous studies with psychedelics could be related to drug doses (low/high, single/multiple) and characteristics of the volunteers (age, healthy/clinical sample) and of the experimental context (use/lack of meditation and specific preparation). Indeed, in some of the psilocybin trials volunteers were previously engaged in spiritual/religious activities (3, 4) or the context included a meditation program and “other practices that emphasized the integration of spiritual values in daily life.” These set and setting characteristics could have facilitated the results since volunteers could be more “open” before the trial, and the context might have magnified that trait. In our trials and in the LSD trials such contextual measures were not used. In our trials specifically, guide/facilitator support during the dosing session was minimal and music was not allowed, so that the environment was kept as quiet and neutral as possible. No group required more investigator interaction during the dosing session. We used the same approach in our open-label trial with depressed patients, were we found antidepressant effects (15). Our protocol was planned in this manner since we still do not know what is the role/effect that different contexts and psychotherapeutic techniques have on the overall and therapeutic effects of psychedelics. Thus, it is still not clear if a more standardized preparation/integration would modify the observed results, especially if we consider the (also) inconsistent results with LSD and psilocybin. However, our setting could have impacted the subjective quality of the acute ayahuasca experience and, maybe, later changes in personality. This should be further explored in future well-powered trials. Finally, regarding age, participants in study 1 were older than in study 2, which could have also contributed to different baseline Openness scores. Despite the above-mentioned limitations, our trials were the first controlled studies to assess the effects of ayahuasca on personality. Further research is needed in larger samples, with enough for item analysis, to better explore the impact of psychedelics like ayahuasca on traits like openness and its facets.

## Conclusion

Compared to baseline, the association of placebo plus ayahuasca in study 2 increased Openness scores 21 days after drug intake, replicating previous findings with LSD and psilocybin. These personality changes could have implications for and be of bigger magnitude in clinical samples, such as patients with depression and anxiety. Further trials with clinical samples are needed to better understand the effects of ayahuasca and other serotonergic hallucinogens on personality.

## Data Availability Statement

The original contributions presented in the study are included in the article/Supplementary Material, further inquiries can be directed to the corresponding author/s.

## Ethics Statement

The studies involving human participants were reviewed and approved by the Research Ethics Committee of the Hospital das Clínicas, Faculty of Medicine of Ribeirão Preto, University of São Paulo (HC-FMRP-USP). All procedures were conducted in accordance with the Declaration of Helsinki and the ethical standards of the Ministry of Health (Resolution No. 466/12 of the National Health Council). The patients/participants provided their written informed consent to participate in this study.

## Author Contributions

FO, JCH, and RS contributed to conception and design of the study. JM, GR, and JB organized the database. JB performed the statistical analysis. JM, GR, and RS wrote the first draft of the manuscript. GS, MY, EC, and MQ performed the chemical analyses of the ayahuasca samples. All authors contributed to manuscript revision, read, and approved the submitted version.

## Conflict of Interest

The authors declare that the research was conducted in the absence of any commercial or financial relationships that could be construed as a potential conflict of interest.

## Publisher's Note

All claims expressed in this article are solely those of the authors and do not necessarily represent those of their affiliated organizations, or those of the publisher, the editors and the reviewers. Any product that may be evaluated in this article, or claim that may be made by its manufacturer, is not guaranteed or endorsed by the publisher.
