# Supplementary material for: Effects of Ayahuasca on Personality: Results of Two Randomized, Placebo-Controlled Trials in Healthy Volunteers
Source: Front Psychiatry. 2021 Aug 6;12:688439. doi: 10.3389/fpsyt.2021.688439 (PMC8377499; doi:10.3389/fpsyt.2021.688439)
Supplement: Supplementary file 1 [file Data_Sheet_1.docx]

Table S1. Eye movement measurements of HCs and MDD subjects.

|  | HCs (n=400) | | | | | |  | MDD (n=37) | | | | | | |
| --- | --- | --- | --- | --- | --- | --- | --- | --- | --- | --- | --- | --- | --- | --- |
|  | naked eye  (n=189) | | glasses  (n=124) | | soft contact lens  (n=87) | |  | naked eye  (n=20) | | glasses  (n=14) | | soft contact lens  (n=3) | | |
|  | median | SD | median | SD | median | SD |  | median | SD | median | SD | median | SD | |
| **Free-viewing test** |  |  |  |  |  |  |  |  |  |  |  |  |  | |
| Number of fixations | 23.00 | 3.27 | 23.00 | 3.40 | 22.50 | 3.25 |  | 21.25 | 4.09 | 23.25 | 6.36 | 21.50 | 3.50 | |
| Duration of fixation | 253.50 | 44.30 | 250.75 | 44.40 | 262.50 | 52.49 |  | 275.38 | 61.24 | 252.75 | 120.18 | 276.50 | 42.17 | |
| Number of saccades | 21.50 | 3.64 | 20.75 | 4.03 | 20.50 | 3.71 |  | 19.75 | 4.58 | 21.50 | 6.64 | 20.00 | 3.97 | |
| Duration of saccades* | 43.00 | 5.83 | 41.38 | 5.48 | 42.25 | 4.74 |  | 46.63 | 4.98 | 40.25 | 8.37 | 42.50 | 2.47 | |
| Saccade amplitude* | 4.14 | 1.09 | 3.91 | 1.28 | 3.94 | 1.18 |  | 3.72 | 0.82 | 3.25 | 1.25 | 3.77 | 0.68 | |
| Average saccade velocity | 94.93 | 17.12 | 90.32 | 21.42 | 92.75 | 20.27 |  | 80.05 | 16.99 | 77.31 | 19.77 | 87.99 | 8.96 | |
| Peak saccade velocity | 188.52 | 42.49 | 188.30 | 46.71 | 166.82 | 44.29 |  | 190.35 | 40.36 | 174.26 | 50.31 | 176.19 | 36.03 | |
| Scanpath length | 111.79 | 27.59 | 110.50 | 30.66 | 108.94 | 28.51 |  | 92.11 | 30.17 | 83.84 | 44.14 | 114.98 | 8.38 | |
| Fixation density | 0.87 | 0.38 | 0.95 | 0.39 | 0.87 | 0.43 |  | 0.93 | 0.36 | 1.11 | 0.66 | 0.58 | 0.17 | |
| Main sequence *v_max_** | 422.82 | 92.73 | 460.81 | 130.81 | 439.38 | 146.02 |  | 409.60 | 99.36 | 469.73 | 113.29 | 416.16 | 130.35 | |
| Main sequence *s* | 8.93 | 3.59 | 9.23 | 5.00 | 10.20 | 4.86 |  | 7.14 | 3.80 | 8.38 | 3.95 | 9.42 | 4.78 | |
| Main sequence *v_0_** | 34.96 | 10.06 | 33.58 | 10.47 | 32.20 | 8.43 |  | 33.52 | 10.48 | 31.53 | 11.28 | 34.71 | 3.95 | |
| Number of blinks | 1.00 | 1.26 | 1.00 | 1.79 | 1.00 | 1.46 |  | 1.00 | 1.57 | 0.75 | 0.80 | 1.00 | 0.58 | |
| **Smooth pursuit test** |  |  |  |  |  |  |  |  |  |  |  |  |  | |
| Horizontal SNR* | 2.04 | 0.16 | 2.06 | 0.16 | 1.98 | 0.14 |  | 2.05 | 0.18 | 1.97 | 0.16 | 2.14 | 0.26 | |
| Horizontal position gain* | 1.01 | 0.02 | 1.01 | 0.03 | 1.02 | 0.03 |  | 1.00 | 0.02 | 1.02 | 0.04 | 1.01 | 0.03 | |
| Horizontal RMSE* | 8.11 | 4.40 | 8.18 | 3.88 | 9.68 | 4.00 |  | 7.84 | 4.60 | 9.98 | 3.69 | 10.72 | 3.12 | |
| Vertical SNR* | 1.85 | 0.21 | 1.89 | 0.19 | 1.77 | 0.17 |  | 1.84 | 0.20 | 1.74 | 0.18 | 1.78 | 0.32 | |
| Vertical position gain* | 0.95 | 0.06 | 0.95 | 0.07 | 0.98 | 0.08 |  | 0.95 | 0.10 | 0.95 | 0.10 | 0.95 | 0.02 | |
| Vertical RMSE* | 13.17 | 7.82 | 13.27 | 8.36 | 15.91 | 7.40 |  | 13.59 | 10.20 | 14.82 | 10.00 | 13.83 | 4.64 | |
| Number of fixations* | 56.50 | 12.13 | 58.25 | 15.50 | 65.00 | 13.09 |  | 61.00 | 11.76 | 55.25 | 11.31 | 76.00 | 34.32 | |
| Duration of fixations* | 271.50 | 69.61 | 251.88 | 66.93 | 227.50 | 61.30 |  | 248.50 | 71.98 | 298.75 | 70.54 | 208.75 | 179.77 | |
| Number of saccades* | 54.50 | 13.50 | 54.50 | 18.21 | 65.00 | 14.51 |  | 60.00 | 11.62 | 52.00 | 11.19 | 76.00 | 33.72 | |
| Duration of saccades | 31.00 | 6.12 | 28.50 | 5.54 | 29.50 | 4.22 |  | 35.00 | 10.67 | 34.63 | 8.17 | 29.00 | 4.92 | |
| Saccade amplitude* | 2.09 | 0.65 | 1.78 | 0.61 | 1.93 | 0.51 |  | 2.39 | 0.71 | 2.63 | 0.74 | 2.02 | 0.45 | |
| Average saccade velocity* | 68.24 | 11.56 | 63.28 | 11.09 | 62.68 | 11.34 |  | 66.74 | 10.76 | 72.88 | 9.08 | 70.91 | 6.63 | |
| Peak saccade velocity* | 112.45 | 38.15 | 93.04 | 39.33 | 79.13 | 30.10 |  | 137.91 | 44.86 | 150.21 | 49.48 | 102.46 | 9.65 | |
| Horizontal velocity gain | 0.84 | 0.11 | 0.87 | 0.11 | 0.85 | 0.09 |  | 0.78 | 0.13 | 0.78 | 0.16 | 0.76 | 0.18 | |
| Vertical velocity gain | 0.74 | 0.13 | 0.78 | 0.13 | 0.79 | 0.12 |  | 0.70 | 0.15 | 0.70 | 0.18 | 0.60 | 0.19 | |
| Number of blinks* | 1.00 | 2.53 | 1.50 | 5.97 | 1.00 | 2.42 |  | 1.75 | 2.99 | 0.50 | 2.01 | 0.50 | 1.32 | |
| **Fixation stability test** |  |  |  |  |  |  |  |  |  |  |  |  |  | |
| Number of fixations* | 2.75 | 2.09 | 3.63 | 2.80 | 2.00 | 2.46 |  | 3.13 | 2.30 | 5.50 | 3.31 | 2.50 | 2.74 | |
| Duration of fixation* | 2020.13 | 1442.88 | 1591.50 | 1413.48 | 3210.25 | 1476.82 |  | 1448.51 | 1269.29 | 1150.44 | 2010.19 | 1774.38 | 1318.03 | |
| Number of saccades* | 1.75 | 2.00 | 2.25 | 2.48 | 0.75 | 2.52 |  | 1.75 | 2.25 | 4.63 | 3.02 | 1.50 | 1.76 | |
| Scanpath length* | 1.04 | 1.93 | 1.47 | 2.59 | 0.66 | 2.08 |  | 1.27 | 1.97 | 1.66 | 2.94 | 1.52 | 3.52 | |
| Number of microsaccades* | 6.75 | 3.35 | 5.00 | 2.87 | 7.75 | 3.75 |  | 6.63 | 4.09 | 6.38 | 4.19 | 5.75 | 5.53 | |
| Number of blinks* | 0.00 | 0.47 | 0.00 | 1.58 | 0.00 | 0.34 |  | 0.00 | 0.58 | 0.00 | 0.80 | 0.25 | 1.11 | |
| RMSE, Root mean square error; SNR, signal-to-noise ratio.  *Represents significant difference in eye conditions in HCs analyzed by analysis of covariance with age as a covariant (corrected by false discovery rate). | | | | | | | | | | | | | |  |

Table S2. Results of t-tests between age-matched groups.

|  | HC (n=37) | | MDD (n=37) | |  | Statistics | | | |  |
| --- | --- | --- | --- | --- | --- | --- | --- | --- | --- | --- |
|  | median | SD | median | SD |  | *df* | t-value | *p*-value | Cohen's *d* |  |
| **Free-viewing test** |  |  |  |  |  |  |  |  |  |  |
| Number of fixations | 23.50 | 2.86 | 21.50 | 4.93 |  | 72 | 1.3 | 0.19 | 0.31 |  |
| Duration of fixation | 251.25 | 37.06 | 267.00 | 86.41 |  | 72 | -2.6 | 0.01 | -0.61 |  |
| Number of saccades | 21.00 | 4.16 | 20.00 | 5.33 |  | 72 | 0.2 | 0.84 | 0.05 |  |
| Duration of saccades | 42.75 | 5.68 | 42.50 | 6.65 |  | 72 | -0.6 | 0.57 | -0.13 |  |
| Saccade amplitude | 4.24 | 1.12 | 3.60 | 0.99 |  | 72 | 1.8 | 0.08 | 0.41 |  |
| Average saccade velocity | 93.94 | 16.66 | 81.99 | 17.50 |  | 72 | 2.5 | 0.02 | 0.57 |  |
| Peak saccade velocity | 198.26 | 45.00 | 181.81 | 43.85 |  | 72 | 0.4 | 0.65 | 0.10 |  |
| Scanpath length | 113.58 | 31.99 | 92.56 | 34.98 |  | 72 | 2.6 | 0.01 | 0.60 |  |
| Fixation density | 1.00 | 0.44 | 0.92 | 0.51 |  | 72 | -0.7 | 0.46 | -0.17 |  |
| Main sequence *v_max_* | 445.98 | 95.11 | 419.68 | 107.90 |  | 72 | 0.0 | 0.99 | 0.00 |  |
| Main sequence *s* | 8.47 | 3.56 | 7.63 | 3.95 |  | 72 | 0.5 | 0.59 | 0.12 |  |
| Main sequence *v_0_* | 33.52 | 9.79 | 32.60 | 10.27 |  | 72 | 0.9 | 0.36 | 0.21 |  |
| Number of blinks | 1.00 | 2.41 | 1.00 | 1.31 |  | 72 | 2.2 | 0.03 | 0.50 |  |
| **Smooth pursuit test** |  |  |  |  |  |  |  |  |  |  |
| Horizontal SNR | 2.02 | 0.15 | 2.02 | 0.18 |  | 72 | 0.6 | 0.54 | 0.14 |  |
| Horizontal position gain | 1.02 | 0.02 | 1.00 | 0.03 |  | 72 | 1.1 | 0.26 | 0.26 |  |
| Horizontal RMSE | 8.19 | 3.09 | 8.78 | 4.11 |  | 72 | -1.1 | 0.27 | -0.26 |  |
| Vertical SNR | 1.85 | 0.21 | 1.80 | 0.20 |  | 72 | 0.6 | 0.58 | 0.13 |  |
| Vertical position gain | 0.96 | 0.07 | 0.95 | 0.09 |  | 72 | 1.5 | 0.13 | 0.36 |  |
| Vertical RMSE | 13.59 | 8.92 | 13.83 | 9.69 |  | 72 | 0.0 | 0.96 | -0.01 |  |
| Number of fixations | 58.50 | 15.63 | 60.00 | 13.99 |  | 72 | 0.3 | 0.80 | 0.06 |  |
| Duration of fixations | 255.75 | 75.64 | 250.75 | 79.92 |  | 72 | -1.1 | 0.29 | -0.25 |  |
| Number of saccades | 54.50 | 18.92 | 59.50 | 13.85 |  | 72 | 0.0 | 0.99 | 0.00 |  |
| Duration of saccades | 31.25 | 5.13 | 34.50 | 9.41 |  | 72 | -3.0 | 3.9.E-03 | -0.69 |  |
| Saccade amplitude | 2.07 | 0.63 | 2.38 | 0.71 |  | 72 | -2.6 | 0.01 | -0.60 |  |
| Average saccade velocity | 66.39 | 13.56 | 70.80 | 9.85 |  | 72 | -0.9 | 0.37 | -0.21 |  |
| Peak saccade velocity | 111.19 | 35.82 | 136.46 | 46.52 |  | 72 | -3.3 | 1.5.E-03 | -0.77 |  |
| Horizontal velocity gain | 0.85 | 0.11 | 0.77 | 0.14 |  | 72 | 2.3 | 0.03 | 0.53 |  |
| Vertical velocity gain | 0.76 | 0.15 | 0.70 | 0.16 |  | 72 | 1.8 | 0.08 | 0.41 |  |
| Number of blinks | 2.00 | 5.65 | 1.00 | 2.57 |  | 72 | 1.7 | 0.10 | 0.39 |  |
| **Fixation stability test** |  |  |  |  |  |  |  |  |  |  |
| Number of fixations | 3.50 | 3.32 | 3.25 | 2.71 |  | 72 | -0.1 | 0.93 | -0.02 |  |
| Duration of fixation | 1719.88 | 1372.79 | 1422.50 | 1558.35 |  | 72 | 0.1 | 0.92 | 0.02 |  |
| Number of saccades | 2.00 | 2.45 | 2.00 | 2.53 |  | 72 | -1.0 | 0.33 | -0.23 |  |
| Scanpath length | 1.70 | 2.77 | 1.36 | 2.43 |  | 72 | -0.1 | 0.96 | -0.01 |  |
| Number of microsaccades | 6.50 | 3.48 | 6.50 | 4.17 |  | 72 | 0.0 | 0.97 | -0.01 |  |
| Number of blinks | 0.00 | 2.39 | 0.00 | 0.70 |  | 72 | 1.3 | 0.21 | 0.29 |  |
| RMSE, Root mean square error; SNR, signal-to-noise ratio. | | | | | | | | | | |
