# Supplementary material for: Effects of Ayahuasca on Personality: Results of Two Randomized, Placebo-Controlled Trials in Healthy Volunteers
Source: Front Psychiatry. 2021 Aug 6;12:688439. doi: 10.3389/fpsyt.2021.688439 (PMC8377499; doi:10.3389/fpsyt.2021.688439)
Supplement: Supplementary file 2 [file Data_Sheet_2.docx]

**Online Resource 2.** Sociodemographic characteristics of volunteers.

|  | **Study 1** | | | **Study 2** | | |
| --- | --- | --- | --- | --- | --- | --- |
| **Sociodemographic Variables** | **Placebo**  **(n = 10)** | **Ayahuasca (n = 10)** | ***p*-values** | **Placebo**  **+ayahuasca (n= 8)** | **CBD**  **+ayahuasca (n=9)** | ***p*-values** |
| **Mean Age (Years) ± Standard Deviation** | 33.4 ± 10.8 | 30.2 ± 10.3 | ns | 25.3 ± 5.41 | 25.3 ± 4.12 | ns |
| **Gender (Females)** | 50% | 70% | ns | 62.50% | 66% | ns |
| **Marital Status** |  |  | ns |  |  | ns |
| **Single** | 80% | 90% |  | 75% | 100% |  |
| **Married** | 20% | 10% |  | 25% | 0% |  |
| **Mean Weigh (Kg) ± Standard Deviation** | 76 ± 13.0 | 69 ± 15.4 | ns | 70.4 ± 19.8 | 66.1 ± 13.3 | ns |
| **Education** |  |  | ns |  |  | ns |
| **Incomplete Secondary Education** | 20% | 30% |  | 25% | 0% |  |
| **Complete Secondary Education** | 30% | 20% |  | 50% | 55% |  |
| **Postgraduate** | 50% | 50% |  | 25% | 45% |  |
| **Professional Occupation** |  |  | ns |  |  | ns |
| **Student** | 10% | 40% |  | 62.5% | 66% |  |
| **Government Employee** | 50% | 10% |  | 12.5% | 0% |  |
| **Self-Employed** | 20% | 0% |  | 12.5% | 11% |  |
| **Other** | 20% | 50% |  | 12.5% | 22% |  |
| **Family Income** |  |  | ns |  |  | ns |
| **1,045–3,135 Reais (approx. 190–570 Dollars)** | 20% | 20% |  | 0% | 0% |  |
| **3,135–6,270 Reais (approx. 570–1,140 Dollars)** | 50% | 40% |  | 87.5% | 55% |  |
| **>6,270 Reais (approx. 1,140 Dollars)** | 30% | 40% |  | 12.5% | 45% |  |
| **Substance Use** |  |  |  |  |  | ns |
| **Cannabis** | - | - |  | 62.5% | 55% |  |
| **Cocaine** | - | - |  | 0% | 11% |  |
| **MDMA (Ecstasy)** | - | - |  | 37.5% | 11% |  |
| **LSD** | - | - |  | 0% | 11% |  |
| **Ayahuasca** | - | - |  | 25% | 11% |  |
| **Solvents** | - | - |  | 0% | 22% |  |

ns: non-significant (*p* > 0.05). *p*-values refer to within-trial differences, not between trials.
