# Supplementary material for: Effects of Ayahuasca on Personality: Results of Two Randomized, Placebo-Controlled Trials in Healthy Volunteers
Source: Front Psychiatry. 2021 Aug 6;12:688439. doi: 10.3389/fpsyt.2021.688439 (PMC8377499; doi:10.3389/fpsyt.2021.688439)
Supplement: Supplementary file 3 [file Data_Sheet_3.docx]

**Online Resource 3.** Mean (SD) NEO-FFI scores in study 1.

| **NEO** | | | | | | |
| --- | --- | --- | --- | --- | --- | --- |
| **Time** | **Neuroticism** | | **Extraversion** | | **Openness** | |
|  | PLA | AYA | PLA | AYA | PLA | AYA |
| **Baseline** | 18.62 (7.05) | 19.14 (9.79) | 31.63 (3.54) | 29.14 (8.23) | 32.88 (2.41) | 32.57 (5.74) |
| **Day 21** | 17.88 (7.45) | 21.14 (6.72) | 33.63 (4.27) | 27.86 (7.27) | 33.13 (2.80) | 33.29 (5.65) |
|  | **Agreeableness** | | **Conscientiousness** | |  |  |
|  | PLA | AYA | PLA | AYA |  |  |
| **Baseline** | 32.25 (2.96) | 33.43 (2.99) | 33.38 (5.39) | 31.43 (5.29) |  |  |
| **Day 21** | 32.5 (3.66) | 33.71 (4.31) | 34.25 (5.01) | 32.29 (5.47) |  |  |

PLA: placebo group; AYA: ayahuasca group.
