# Supplementary material for: Effects of Ayahuasca on Personality: Results of Two Randomized, Placebo-Controlled Trials in Healthy Volunteers
Source: Front Psychiatry. 2021 Aug 6;12:688439. doi: 10.3389/fpsyt.2021.688439 (PMC8377499; doi:10.3389/fpsyt.2021.688439)
Supplement: Supplementary file 4 [file Data_Sheet_4.docx]

**Online Resource 4.** Mean (SD) NEO-FFI scores in study 2.

| **NEO** | | | | | | |
| --- | --- | --- | --- | --- | --- | --- |
| **Time** | **Neuroticism** | | **Extraversion** | | **Openness** | |
|  | PLA+AYA | CBD+AYA | PLA+AYA | CBD+AYA | PLA+AYA | CBD+AYA |
| **Baseline** | 17.80 (7.40) | 21.25 (3.69) | 31.60 (3.65) | 32.25 (4.65) | 31.60 (4.45) | 37.75 (4.92) |
| **Day 21** | 17.60 (6.23) | 22.25 (6.02) | 32.00 (4.36) | 32.88 (4.39) | 36.40 (5.86) | 37.38 (6.78) |
|  | **Agreeableness** | | **Conscientiousness** | |  |  |
|  | PLA+AYA | CBD+AYA | PLA+AYA | CBD+AYA |  |  |
| **Baseline** | 33.80 (8.56) | 34.38 (4.72) | 31.20 (8.44) | 33.88 (3.76) |  |  |
| **Day 21** | 35.40 (11.80) | 34.25 (4.40) | 32.20 (8.04) | 32.50 (4.11) |  |  |

PLA+AYA: placebo plus ayahuasca group; CBD+AYA: cannabidiol plus ayahuasca group.
