# Supplementary figures and images for: Effects of Ayahuasca on Personality: Results of Two Randomized, Placebo-Controlled Trials in Healthy Volunteers
Source: Front Psychiatry. 2021 Aug 6;12:688439. doi: 10.3389/fpsyt.2021.688439 (PMC8377499; doi:10.3389/fpsyt.2021.688439)

**Online Resource 1.** Details of participant screening and inclusion in the trials.


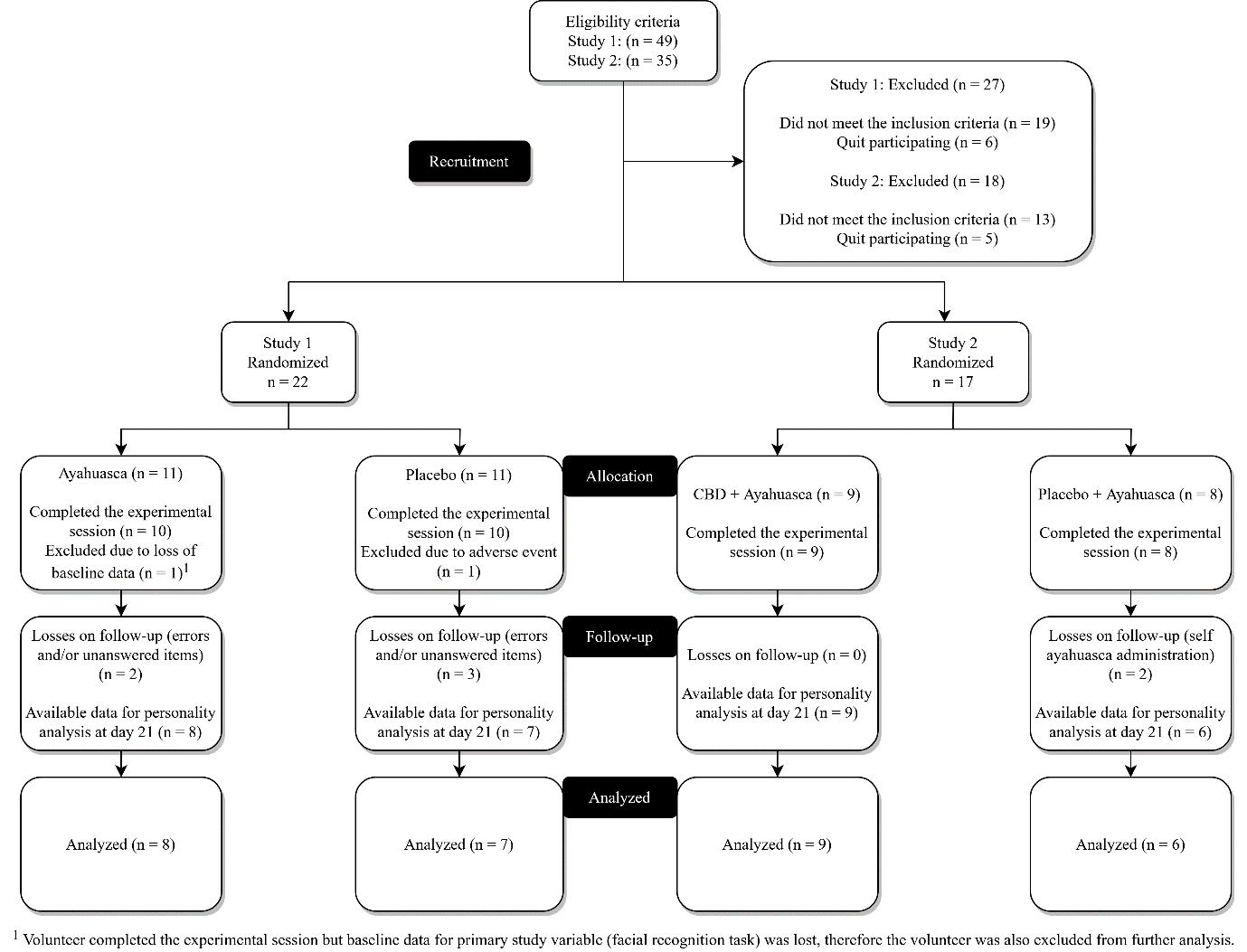

Supplement: Supplementary file 5 [file Table_1.DOCX]
